# Cannabinoidhyperemesis als Differenzialdiagnose von Übelkeit und Erbrechen in der Notaufnahme

**DOI:** 10.1007/s00101-020-00850-2

**Published:** 2020-10-22

**Authors:** F. Korn, S. Hammerich, A. Gries

**Affiliations:** Zentrale Notaufnahme, Universitätsklinikum Leipzig, Liebigstraße 4, 04103 Leipzig, Deutschland

**Keywords:** Zyklisches Erbrechen, Cannabiskonsum, Drogen, Therapierefraktäres Erbrechen, Unklares Erbrechen, Cyclic vomiting, Cannabis abuse, Drugs, Therapy resistent vomiting, Unclear vomiting

## Abstract

Das Cannabinoid-Hyperemesis-Syndrom (CHS) ist ein durch regelmäßigen Cannabiskonsum verursachtes Krankheitsbild, dass durch zyklische Episoden von starkem Erbrechen gekennzeichnet ist. Klassische Antiemetika bringen oft keine Linderung, teilweise wurden auch letale Verläufe beschrieben. In dem Fall eines jungen Mannes konnte nach 4 Vorstellungen die Diagnose gestellt werden. Zu diesem Zeitpunkt hatte der Patient bereits eine Abdomen-CT sowie eine Gastroskopie erhalten. Bei eingehaltener Cannabiskarenz zeigte sich 6 Monate nach Vorstellung ein kompletter Rückgang der Symptomatik.

## Abstract (en)

Cannabinoid hyperemesis syndrome (CHS) is caused by regular cannabis consumption and marked by cyclic episodes of severe emesis, often years after the beginning of cannabis consumption. Classical antiemetic treatment often shows no effect and fatal outcomes have sometimes also been reported. This article describe the case of a young man in whom the diagnosis was made after four presentations due to the typical anamnesis. At this point in time he had already undergone an abdominal computed tomography (CT) and gastroscopy, both with no pathological findings. The symptoms completely receded 6 months after presentation by maintaining cannabis abstinence.

## Anamnese

Ein 31-jähriger männlicher Patient stellte sich mit stärkstem Erbrechen und Oberbauchschmerzen und in deutlich reduziertem Allgemeinzustand vor. Bereits 3 Vorstellungen mit gleicher Symptomatik innerhalb von 3 Monaten waren erfolgt. Die Beschwerden hätten nach einer Israelreise vor 3 Monaten begonnen, insgesamt habe er bereits 8 kg Gewicht verloren. Ein klarer Auslöser war nicht zu eruieren. Der Stuhlgang sei regelrecht, ein Zusammenhang mit der Nahrungsaufnahme bestand ebenfalls nicht, Fieber oder anderweitige Beschwerden wurden verneint. Beruflich ist der Patient als Chemiker mit organischen Stoffen tätig. In der Genussmittelanamnese wurde ein Konsum von 15 Zigaretten sowie ein langjähriger, mehrfach wöchentlicher Cannabiskonsum angegeben. An Vordiagnosen bestanden eine Depression, welche mit Escitalopram 20 mg einmal täglich behandelt wurde, sowie ein Zustand nach Appendektomie. Allergien seien nicht bekannt. Bei der aktuellen Vorstellung gab der Patient an, dass heiße Bäder die Beschwerden lindern würden.

## Befund

**Figure 1:**
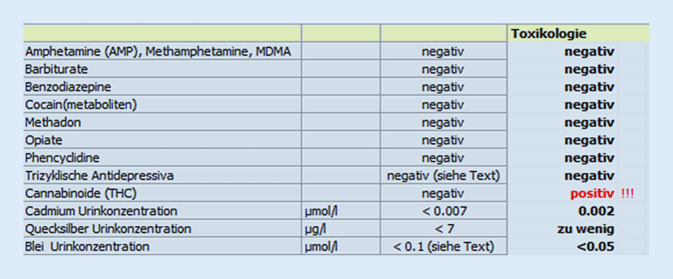

In den 4 klinischen Untersuchungen präsentierte sich jeweils ein Patient mit stabilen Vitalparametern, teilweise mit diskretem epigastrischem Druckschmerz ohne Abwehrspannung. Laborchemisch zeigten sich außer einer am ehesten emesisbedingten Leukozytose (16,2 [3,5–9,8] × 10^9^/l) keine weiteren Auffälligkeiten Das toxikologische Screening war positiv für Tetrahydrocannabinol (THC); Schwermetallverbindungen konnten nicht nachgewiesen werden (Abb. 1). Beim initialen Besuch waren aufgrund des starken, nichtstillbaren Erbrechens eine Sonographie und Computertomographie des Abdomens durchgeführt worden. Beide Untersuchungen sowie eine ambulant durchgeführte Gastroskopie erbrachten jedoch Normalbefunde. Nach infektiologischer Rücksprache war eine infektiöse Ursache bei rein emetischen Beschwerden als unwahrscheinlich anzusehen.

## Diagnose

Aufgrund des angegebenen Cannabiskonsums, der auf Nachfrage berichteten Linderung der Beschwerden durch heiße Bäder und der erfolgten Voruntersuchungen ohne pathologische Befunde wurde ein Cannabinoid-Hyperemesis-Syndrom (CHS) diagnostiziert und eine strikte Cannabiskarenz empfohlen.

## Therapie und Verlauf

Während der Vorstellungen zeigten klassische Antiemetika nur eine unzureichende Wirkung, so erhielt der Patient während der ersten Vorstellung kumulativ i.v. 10 mg Metoclopramid, 3 mg Granisetron, 4 mg Dexamethason sowie 5 mg Haloperidol. Während der folgenden Vorstellung kam es zu rezidivierendem Erbrechen trotz Gabe von multiplen Antiemetika u. a. Ondansetron‑/Granisetron. In einer Nachkontrolle 6 Monate nach Empfehlung zur Cannabiskarenz berichtete der Patient, bei eingehaltener Karenz keine weitere Emesisepisoden erlitten zu haben. Ferner habe er 8 kg Gewicht, die er verloren hatte, wieder zugenommen.

## Diskussion

Das CHS wurde erstmalig 2004 in einer Fallserie mit 9 Patienten beschrieben. In den USA wird von einzelnen Autoren von bis zu 2,75 Mio. Patienten ausgegangen, die an CHS leiden [2]. Nach der Legalisation von Cannabis in Colorado wurde eine Verdoppelung der CHS-Inzidenz beschrieben [4].

In einer 2012 von Simonetto et al. veröffentlichten Fallserie mit 98 Patienten wurden Diagnosekriterien vorgeschlagen, welche die Diagnostik erleichtern sollen (Tab. 1) [9]. Eine Metaanalyse mit insgesamt 221 Fällen von Soerensen et al. bestätigte diese Diagnosekriterien [10]. Weiterhin typisch sind ein schlechtes Ansprechen auf Antiemetika sowie der Beginn teilweise erst Jahre nach Beginn des Cannabiskonsums [10]. In Einzelfällen wurden tödliche Ausgänge beobachtetet: So wurden in einem 2019 veröffentlichten Review 2 Fälle beschrieben, worin es aufgrund von prärenalem Nierenversagen und Elektrolytstörungen zum Exitus letalis kam [7]. Somit ist therapeutisch auf eine ausreichende Rehydration sowie Elektrolytsubstitution zu achten. Hinsichtlich des Erbrechens zeigt topisch appliziertes Capsaicin über eine Interaktion des TRVP-1-Rezeptors, der auch bei Umgebungshitze, wie z. B. bei heißen Bädern, aktiviert wird, eine gute Wirkung [6]. Es sollten dabei stärkere Konzentrationen um 0,075 % verwendet werden; die Creme sollte v. a. am Körperstamm aufgetragen werden; der Kontakt mit Schleimhäuten und dem Genitalbereich ist zu vermeiden. Als Nebenwirkung können lokale Hautreaktionen auftreten. Ferner wurden in Fallberichten Therapieerfolge mit Benzodiazepinen und Antipsychotika beschrieben (Tab. 2; [6]). Langfristig ist als einzige kurative Maßnahme die Cannabiskarenz anzusehen [10].

**Table 1:** 

*Essenziell:* langjähriger Cannabiskonsum
*Hauptkriterien*
Schwere Übelkeit/Erbrechen
Regredienz bei Cannabiskarenz
Besserung durch heißes Baden/Duschen
Epigastrische/periumbilikale Schmerzen
Mindestens wöchentlicher Cannabiskonsum
*Nebenkriterien*
Alter <50 Jahre
>5 kg Gewichtsverlust
Primär morgendliche Symptome
Normales Stuhlverhalten
Unauffällige Labor‑, Endoskopie‑, Radiologiebefunde

**Table 2:** 

Therapeutische Optionen
1. Wahl: Capsaicinsalbe, 0,075 %ig, am Körperstamm/heiß duschen
Alternativen:
Benzodiazepine: z. B. 1 mg Lorazepam oder 5–10 mg Diazepam i.v.
Antipsychotika: z. B. 5 mg Haloperidol oder Olanzapin i.m.
*Cave;* oft kein Ansprechen auf „klassische Antiemetika“

Die exakte Pathophysiologie des CHS ist noch unklar. In Cannabis sind insgesamt über 750 chemische und 104 anders wirkende Cannabinoide enthalten. Eine Interaktion dieser Substanzen mit dem endogenen Cannabinoidrezeptor 1 (CR-1), im ZNS und im gastrointestinalen Trakt wird vermutet [3]. Stimulation des CR‑1 führt zu verminderter Gastromotilität, sodass eine Hypothese zur Wirkung auf einer durch Cannabis induzierten Gastroparese mit konsekutivem Erbrechen beruht. In einer Fallserie mit 98 Personen zeigten jedoch nur 30 % aller Patienten eine verzögerte gastrale Entleerung [9]. Eine andere Hypothese stellt eine Gefäßdilatation im Splanchnikusgebiet in den Raum, welche, ähnlich wie beim hepatorenalen Syndrom, Übelkeit verursacht. Heiße Bäder würden zu einer peripheren Gefäßdilatation führen und damit das Gleichgewicht wiederherstellen [8]. Andere Autoren vermuten eine Beeinflussung der cerebrogastrointestinalen Achse, da niedrige Dosen von Cannabiol in Mausversuchen antiemetisch wirkten, erhöhte Dosen jedoch mit verstärkter Übelkeit und Erbrechen einhergingen [5]. Ferner steht eine Beeinflussung durch individuelle genetische Faktoren im Raum, da nur eine Subpopulation von Cannabiskonsumenten entsprechende Symptome entwickelt. Eine aktuelle Studie konnte keine relevanten Unterschiede der THC- und Cannabinoidkonzentrationen im Urin und in den Haaren zwischen CHS-Patienten und asymptomatischen Cannabiskonsumenten feststellen, sodass die Autoren von einem idiosynkratischem Pathomechanismus ausgehen [1].

Ein Problem ist neben der großen Anzahl der bei der Verbrennung von Cannabis entstehenden Cannabinoide auch die große Zahl physiologischer Vorgänge, in die der CR‑1 eingebunden ist. Eine sichere Aussage zu Signalkaskaden ist damit schwierig. Abzugrenzen ist das CHS vom „cyclic vomiting syndrome“ (CVS), das keine Besserung durch heiße Bäder zeigt und nicht mit Cannabiskonsum assoziiert ist.

## Fazit für die Praxis

Das Cannabinoid-Hyperemesis-Syndrome (CHS) ist ein Krankheitsbild mit zyklischem, stärkstem Erbrechen aufgrund von regelmäßigem Cannabiskonsum, das auch noch Jahre nach Erstkonsum auftreten kann. Insgesamt sind noch viele Fragen hinsichtlich Pathophysiologie und Prävalenz offen. Linderung bringen im Akutfall heiße Bäder, Capsaicin, Neuroleptika oder Benzodiazepine sowie, langfristig gesehen, die Cannabiskarenz. Die klassische antiemetische Therapie zeigt oft keinen Erfolg. Nicht zuletzt vor dem Hintergrund des steigenden Cannabiskonsums sollte es als mögliche Differenzialdiagnose bei rezidivierenden Episoden von Erbrechen in Betracht gezogen werden.
